# UAV-UGV-UMV Multi-Swarms for Cooperative Surveillance

**DOI:** 10.3389/frobt.2021.616950

**Published:** 2021-02-19

**Authors:** Daniel H. Stolfi, Matthias R. Brust, Grégoire Danoy, Pascal Bouvry

**Affiliations:** ^1^ Interdisciplinary Centre for Security, Reliability and Trust (SnT), University of Luxembourg, Esch‐sur‐Alzette, Luxembourg; ^2^ FSTM/DCS, University of Luxembourg, Esch‐sur‐Alzette, Luxembourg

**Keywords:** swarm robotics, mobility model, UAV, UGV, UMV, competitive coevolutionary genetic algorithm, surveillance system, parameter optimisation

## Abstract

In this paper we present a surveillance system for early detection of escapers from a restricted area based on a new swarming mobility model called CROMM-MS (Chaotic Rössler Mobility Model for Multi-Swarms). CROMM-MS is designed for controlling the trajectories of heterogeneous multi-swarms of aerial, ground and marine unmanned vehicles with important features such as prioritising early detections and success rate. A new Competitive Coevolutionary Genetic Algorithm (CompCGA) is proposed to optimise the vehicles’ parameters and escapers’ evasion ability using a predator-prey approach. Our results show that CROMM-MS is not only viable for surveillance tasks but also that its results are competitive in regard to the state-of-the-art approaches.

## 1 Introduction

The use of different types of vehicles arranged in swarms is a promising strategy (Girma et al., 2020) which allows to take advantage of the capabilities of each member while minimising their limitations. These heterogeneous swarms are based on collective behaviors of social insects where collaborations emerge to achieve a common goal (Jeong et al., 2019) while avoiding no-fly zones, extending battery life, or braving inclement weather.

Unmanned Aerial Vehicles (UAVs), also known as drones, are widely used in many applications nowadays (McNeal, 2016). In surveillance scenarios (Brust et al., 2017), they can explore different areas at a relatively high speed using their cameras, featuring excellent communication capabilities (Batista da Silva et al., 2017). On the other hand, UAVs have a reduced flight time, cannot carry larger payloads, and have big troubles to stand in a static position when there are strong winds.

Unmanned Ground Vehicles (UGVs) are able to work over a wide variety of terrains, sharing space with humans, searching for targets while avoiding obstacles (Brust and Strimbu, 2015). UGVs are good candidates for a joint usage with UAVs, complementing each other to act in different terrain characteristics (Stolfi et al., 2020c). Although UGVs are slower than UAVs and have a limited communication range (line of sight, obstacles, etc.), they have a higher autonomy and are frequently used to support UAVs providing a moving recharging station and transportation between mission objectives (Waslander, 2013).

Unmanned Surface Vehicles (USVs), which include Unmanned Marine Vehicles (UMVs), have been proposed as critical components of the future naval forces (Yan et al., 2010) to perform missions such as mine countermeasures, maritime security, and maritime interdiction operations support, among others. They are thought to improve existing naval capabilities offering a reduction in both operational time and cost (Costanzi et al., 2020).

Unpredictability is a essential feature when calculating trajectories for robots which have to perform risky missions such as surveillance, patrolling, or searching for mines. Mobility models based on chaotic systems present such desired unpredictability (Iba and Shimonishi, 2011), only being sensitive to the initial conditions and the implementation which can be made via software to be used in a simulator (Rosalie et al., 2018) or implemented as part of the robot electronics, featuring a true random bit generator obtained from a multi-scroll attractor, to provide waypoints for UAVs (Volos et al., 2012).

**FIGURE 1:**
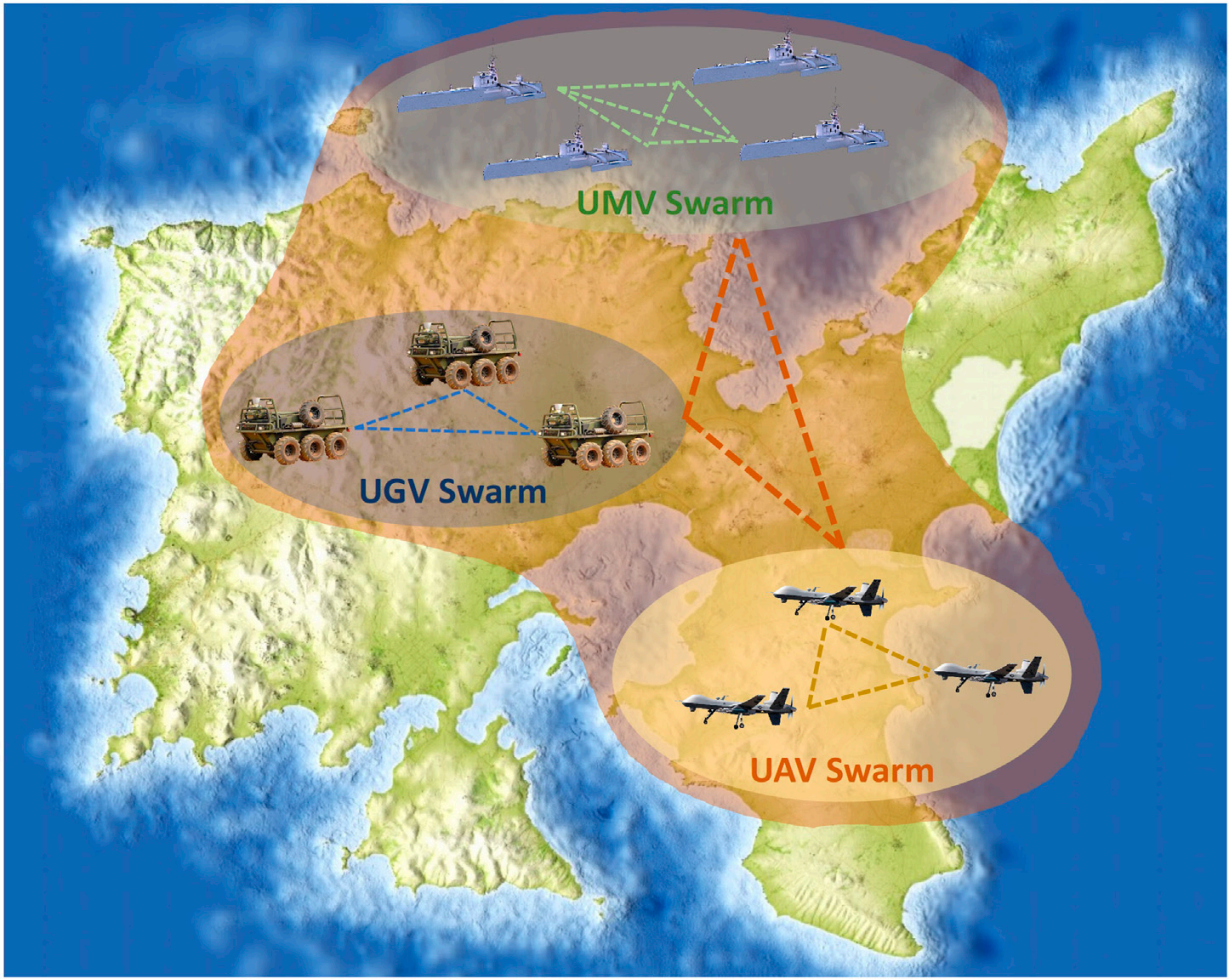
Inter-swarm collaborations as proposed in the HUNTED project – see https://hunted.gforge.uni.lu.

Our proposal consists of a novel multi-swarm surveillance system (Figure 1) where UAVs, UGVs, and UMVs collaborate to achieve early detection of escapers from a restricted area. To this end, we introduce an extension of CROMM (Chaotic Rössler Mobility Model), initially designed for homogeneous vehicles and area coverage, for now addressing heterogeneous multi-swarms and spotting escaper in surveillance scenarios. We aim to exploit the described best features of each vehicle class to achieve better results than homogeneous solutions. The main contributions of this paper are:

1. A new mobility model called CROMM-MS (Chaotic Rössler Mobility Model for Multi-Swarms) to control UAVs trajectories with the aim of maximising early escaper detection.
2. A predator-prey approach to train and improve this surveillance system.
3. A Competitive Coevolutionary Genetic Algorithm (CompGA) specially designed for optimising the vehicles parameters and improving escapers to be valid evaluators.

The remainder of this paper is organised as follows. In the next section, we review the state of the art related to our work. In Section 3 our approach is presented. The experimental results are in Section 4. And finally, Section 5 brings discussion and future work.

## 2 Related Work

Multi-pursuer and multi-evader games have received great attention in the literature. In (Makkapati and Tsiotras, 2019, 2020) two single navigation laws (constant bearing and pure pursuit) are proposed using Apollonius circles and curves. Additionally, a task allocation algorithm is proposed for the pursuers to solve the problem in finite time for any number of pursuers and evaders. A Graph Neural Network trained with Reinforcement Learning was used in (Deka and Sycara, 2020) to obtain complex strategies for two teams of agents. The authors also proposed a mixed cooperative-competitive multi agent environment called FortAttack to test their proposal. After the training process, they achieved highly competitive, emergent heterogeneous behavior between the homogeneous agents. In (Pierson et al., 2017) an algorithm for cooperative pursuit of multiple evaders using multiple pursuers is proposed. It uses a global area minimisation strategy based on the Voronoi tessellation of the environment to capture all the evaders in finite time. Two successful experiments were conducted in a 4x3-metre real environment using four pursuers against four autonomous evaders and also against one human-controlled evader and three autonomous evaders.

Path planning algorithms using chaotic dynamics have been used to get unpredictable trajectories. In (Petavratzis et al., 2019) a modification of the well-known logistic map is used to generate a chaotic pseudorandom bit generator (CPRBG) to produce a bit sequence. This sequence is then used to control a robot which moves by a grid in four or eight different directions. A simple, memory efficient, pheromone model is also proposed to improve the achieved coverage level. In (Moysis et al., 2020) an extension of the previous paper is discussed to avoid the use of a CPRBG taking into account the limited onboard memory and microcontrollers. This new approach is based on getting chaotic tactics by applying a logistic map and a modulo operator. In this article, pheromones are also proposed to increase coverage level and reduce the number of revisits in previous cells.

Coevolutionary algorithms are a good choice to solve problems involving cooperation or competition between different population of individuals. In (Tiguercha et al., 2014) a competitive coevolutionary algorithm is presented to model the interactions of several agents to find the optimal binding strategies in a deregulated electricity market. Each agent is modeled as an adaptive evolutionary agent that acts strategically in order to maximise their profits. A competitive coevolutionary search to the code-smells detection problem is proposed in Boussaa et al. (2013). The authors present two populations that compete one against the other. The first one generates a set of detection rules to maximise the coverage of code-smell examples, while the second population focuses on maximising the number of code smells that cannot be detected. In (Wiegand et al., 2001) an empirical study about cooperative coevolutionary algorithms is presented. Three methods for assigning fitness values based on its collaborations are proposed as well as different collaboration mechanisms using collaborator pools of different sizes.

Several recent research works address cooperative heterogeneous multi-robot systems to perform a variety of tasks (Huang et al., 2019; Rizk et al., 2019). In (Vu et al., 2018) several UAV-UGV cooperation tasks for applications in the field of architecture are presented. Focusing on each vehicle characteristics, UGVs are thought to exploit their working autonomy and high level of interaction with the user while UAVs are more appropriate for communications and tracking support. A probabilistic and scalable new strategy to solve the multi-robot patrol problem is proposed in (Portugal and Rocha, 2016). It uses Bayesian decision-making combined with adaptive learning to achieve intelligent patrolling routes which are tested in a simulation environment as well as in the real world. In (Jayavelu et al., 2018) UGVs are proposed as mobile refuelling and maintenance stations for UAVs. The authors present a framework to calculate the optimal number of UGVs and their location according to the density and position of the moving UAVs. A three-layer surveillance system is proposed in Lee et al., (2019) where UGVs are used as ground-level proximity sensors, UAVs in the second layer use vision sensors, and in the upper layer aerostats (hot-air balloons) provide a broader view of the surveillance area. The authors use an extended DDDAMS (Dynamic-data-driven Adaptive Multi-scale Simulation) using a real-time detection and classification algorithm to predict the target’s location based on a human behavior model. A cooperative exploration solution for search and rescue application in a damaged building is presented in (Hood et al., 2017). It consists of a UGV which navigates through the free indoor space and a UAV providing enhanced situational awareness. In (Arbanas et al., 2018) a decentralised task planning and coordination framework is proposed. It includes a symbiotic aerial vehicle-ground vehicle robotic team where UAVs are used for aerial manipulation tasks, while UGVs assist them by providing safe landing areas and transport. Additionally, UAVs also helps UGVs to negotiate obstacles from their vantage point.

Our proposal does not involve two but three different types of vehicles arranged in nine swarms where each group supports its counterparts performing surveillance tasks. Predators do not have any information about preys and preys are aware of predators only when they are in their vision range. We use a competitive predator-prey approach and optimise not only the autonomous vehicles but also escapers from a restricted area in order to present smarter opponents to be spotted as soon as possible.

## 3 Materials and Methods

We present in this paper an autonomous intelligent surveillance system for detection of escapers breaking out of a restricted area. It is composed by swarms of unmanned autonomous vehicles of different types which patrol the surveillance area in order to spot individuals before they reach the map borders. We follow a predator-prey approach optimising all the entities involved in each escape scenario using an evolutionary bio-inspired technique as described in the following sections.

### 3.1 Swarms of Autonomous Vehicles (Predators)

Our surveillance system is composed of three types of autonomous vehicles each equipped with different detection capabilities:

• UGVs: medium speed, short detection range, 90-degree vision.
• UAVs: high speed, medium detection range, zenithal camera.
• UMVs: low speed, high detection range, 360-degree detection.

We propose CROMM-MS (Chaotic Rössler Mobility Model for Multi-Swarms) an extension of the CROMM (Rosalie et al., 2018) mobility model adapted to heterogeneous multi-swarms of unmanned vehicles moving throughout large scenarios. CROMM is a pure chaotic mobility model where the mobility decisions are taken according to the first return map of a Rössler system (Rössler, 1976). The first return map provides an unpredictable sequence of values ρ∈[0−1], that are used to decide the next moving direction of a vehicle following a probability partition. Consequently, if ρ<1/3, the vehicle turns right (−π/4); if 1/3≤ρ<2/3, the vehicle turns left (π/4); and if ρ≥2/3, it keeps moving ahead.

**FIGURE 2:**
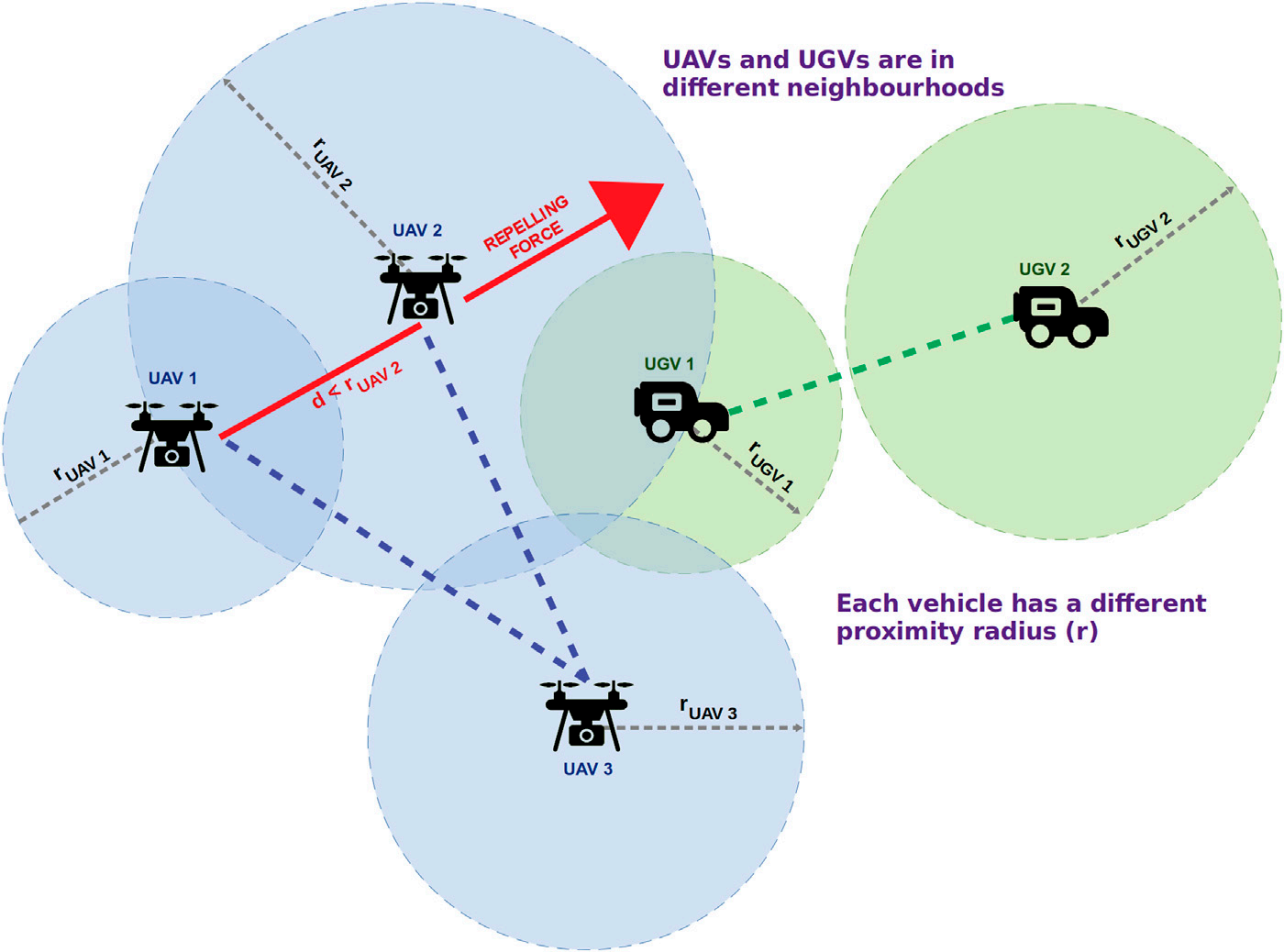
Chaotic Rössler Mobility Model for Multi-Swarms (CROMM-MS). Vehicles have a different proximity radius r to control the swarm movement.

Unlike CROMM, when there are other vehicles in the neighborhood given by the proximity radius *r*, our approach uses a repelling vector calculated taking into account the other vehicles, to decide the next moving direction as shown in Figure 2. If there are no other vehicles in the neighborhood, CROMM-MS works as a pure chaotic mobility model (like CROMM) where the mobility decisions are taken according to the first return map ρ. CROMM-MS runs in each vehicle *i* using a different proximity radius $r_{i}$ to be optimised taking into account each vehicle’s characteristics, with the aim of better spreading the swarms’ members throughout the surveillance scenario, improving coverage and detection rates, without creating big gaps that could be exploited by escapers.

Algorithm 1 shows the pseudocode of CROMM-MS. First, the current angle for each vehicle in the swarm respect to the others is obtained, and its proximity radius *r* is checked against the rest of vehicles of the same type. If some vehicles are closer than *r*, i.e. V≠∅, the new moving direction (angle) is calculated according to the respective repelling forces ($\vec{\Delta }$). Otherwise, pure chaotic is used, based on the value of ρ and the aforementioned probability partition.

Algorithm 1 Pseudocode of CROMM-MS.
**procedure CROMM-MS**
  **for all** u∈U **do** ▷For each vehicle in the swarm
   angle←CurrentAngle(u)
   V←v∈U:v≠u∧Distance(v,u)<r
   **if** V≠∅ **then** ▷If there are vehicles closer than *r*
    $\vec{\Delta }\leftarrow \sum_{v\in V}\left(\vec{u}-\vec{v}\right)$
    $\text{angle}\leftarrow a\tan\left(\Delta_{y}/\Delta_{x}\right)$
   **else** ▷CROMM
    ρ←next value in first return map
    **if** ρ<1/3 **then**
     turn←−π/4 ▷Turn right
    **else if** ρ<2/3 **then**
     turn←π/4 ▷Turn left
    **else**
     turn←0 ▷Go straight on
    **end if**
    angle←angle+turn
   **end if**
   Move(u,angle) ▷The predator moves
  **end for**
 **end procedure**

### 3.2 Escapers (Preys)

We have also designed an escaper (prey) mobility model as a complementary component of our system. It consists of a series of parameters to be optimised simultaneously with the vehicles (predators). Each escaper has an *escape time* ($\epsilon_{t}$) which defines when it breaks out from the facilities in the center of the map and tries to reach one of the *borders* ($\epsilon_{b}$) at a predefined *coordinate* ($\epsilon_{c}$). Finally, the running *speed* ($\epsilon_{s}$) and the intensity of the *avoidance* ($\epsilon_{a}$) manoeuvrers (repulsive force) are also parameterised aiming to present difficult adversaries to our surveillance solution.

**FIGURE 3:**
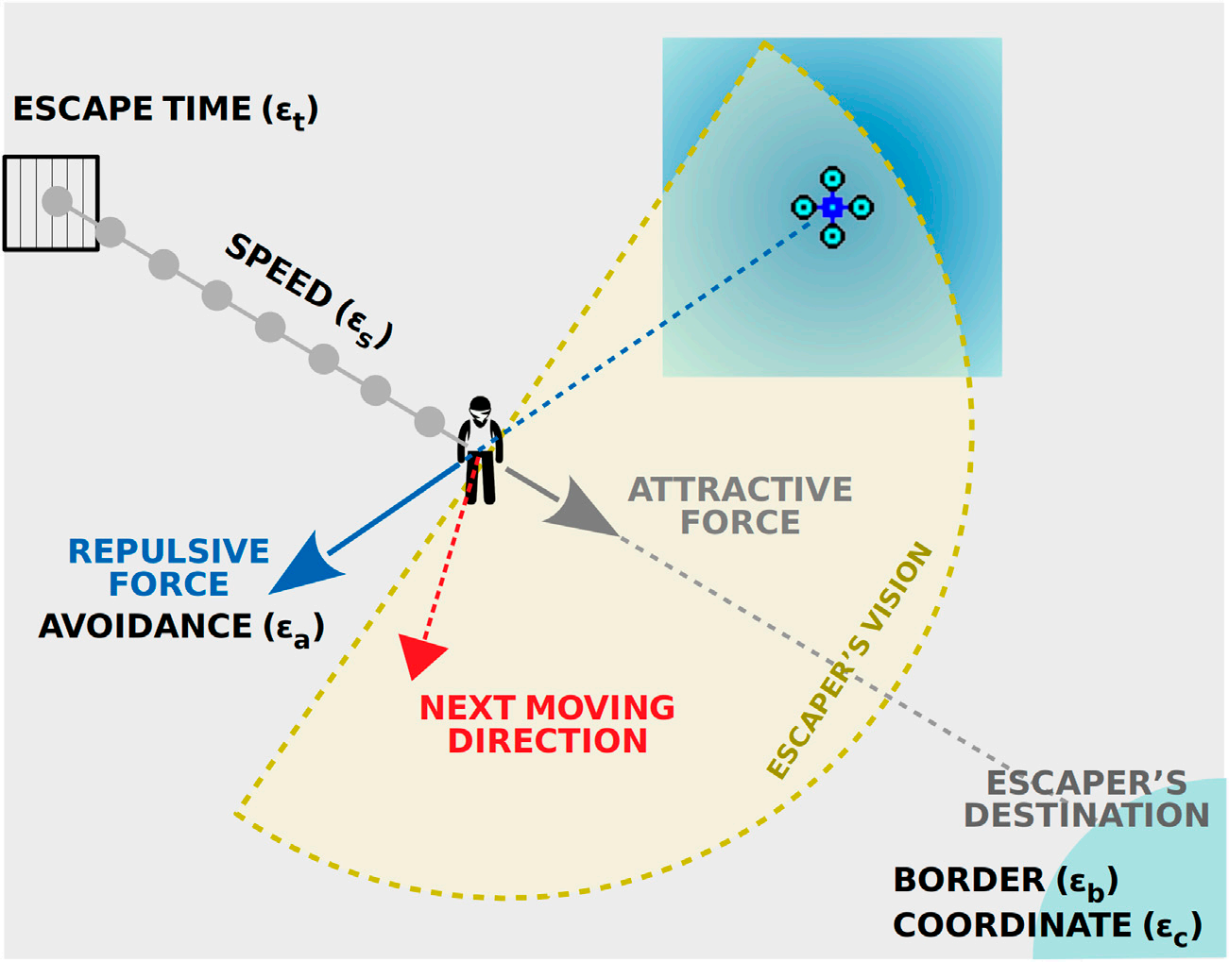
Escaper’s parameters to be optimised for evading patrolling vehicles.

Table 1 shows the parameter list of each escaper and in Figure 3 the forces involved in the escaper’s mobility decision are illustrated. These degrees of freedom provide escapers the ability of deciding when attempting to run away, the less populated region of the map for that escape attempt, and how to react more efficiently to the predators’ menace. All these parameters are optimised by our competitive algorithm as described in the following section.

**TABLE 1:** Parameters of each escaper (prey).

Parameter	Symbol	Units	Values	Type
Border	$\epsilon_{b}$	N,S,E,W	(0−3)	Integer
Coordinate	$\epsilon_{c}$	Coordinate	(0−399)	Integer
Speed	$\epsilon_{s}$	Simulation ticks	(2−6)	Integer
Time	$\epsilon_{t}$	Simulation ticks	(300−1200)	Integer
Avoidance	$\epsilon_{a}$	—	(0.00−2.00)	Real

### 3.3 Competitive Coevolutionary Genetic Algorithm (CompCGA)

We propose a Competitive Coevolutionary Genetic Algorithm (CompCGA) for optimising the proximity radius (*r*) of each autonomous vehicle and the intruders’ parameters in a competitive way following a predator-prey approach. We have taken some initial steps developing our CompCGA in (Stolfi et al., 2020b) optimising a homogeneous swarm of UAVs and a number of intruders in a surveillance scenario. We have now adapted our algorithm and improved it, using two Hall of Fame tables to be sure that the best specimens are used to evaluate their counterparts as well as keeping a memory of the former good candidates. This new version of CompCGA needs more simulations to evaluate an individual but ensures that each fitness value represents how good is a given configuration at defeating several competitors, instead of just using the last best competitor from the other population.

**FIGURE 4:**
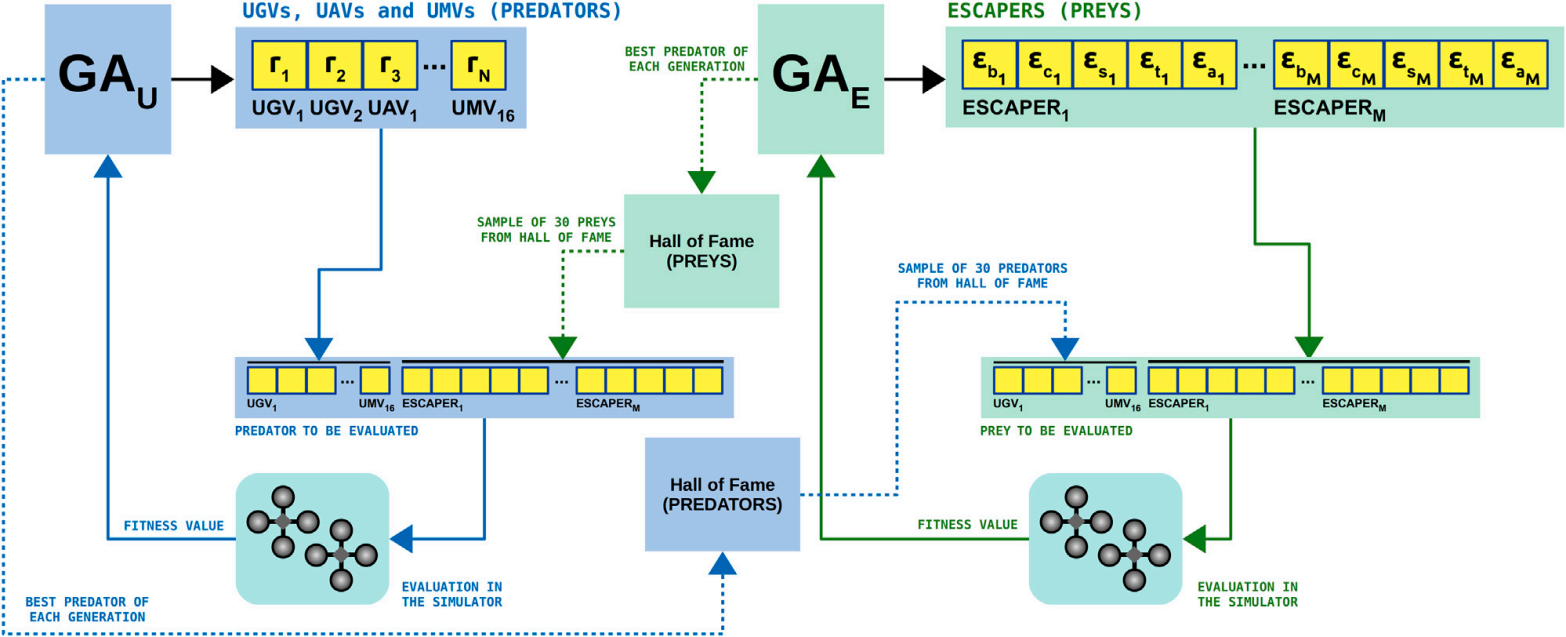
Diagram of the competitive coevolutionary genetic algorithm (CompCGA).

We have used a Genetic Algorithm (GA) to address each parameter set individually as depicted in Figure 4. Genetic Algorithms (Goldberg, 1989; Holland, 1992) are efficient methods for solving combinatorial optimisation problems. They simulate processes present in evolution such as natural selection, gene recombination after reproduction, gene mutation, and the dominance of the fittest individuals over the weaker ones. In this work we consider generational GAs where an offspring of λ individuals is obtained from the population μ, so that the auxiliary population *Q* contains the same number of individuals (20 in our case) as the population *P*. Two identical GAs (GAU for vehicles and GAE for escapers) are used in the CompCGA to perform the coevolution of their populations. The pseudocode of one of these GAs is presented in Algorithm 2.

Algorithm 2 Pseudocode of each Genetic Algorithm (GA).
   **procedure** GA $N_{i},P_{c},P_{m},k$
  t←∅
  Q(0)←∅ ▷Q = auxiliary population
  $P\left(0\right)\leftarrow \text{Initialisation}\left(N_{i}\right)$ ▷P = population
  **while not** TerminationCondition() **do**
   Q(t)←Selection(P(t))
   $Q\left(t\right)\leftarrow \text{Crossover}\left(Q\left(t\right),P_{c}\right)$
   $Q\left(t\right)\leftarrow \text{Mutation}\left(Q\left(t\right),P_{m},k\right)$
   Evaluation(Q(t))
   P(t+1)←Replacement(Q(t),P(t))
   t←t+1
  **end while**
 **end procedure**

After initialising *t* and Q(0), the GA generates the initial population P(0) by using the *Initialisation* function. The main loop is executed while the *TerminationCondition* is not fulfilled (we stop after 20,000 evaluations). Into the main loop, the *Selection* operator is applied to populate Q(t) using Binary Tournament (Goldberg and Deb, 1991). In the following lines, our *Crossover* and *Mutation* operators (Stolfi et al., 2020a, Stolfi et al., 2020d) are applied. The former with the aim of widely exploring the search space and the latter to make small modifications to each individual (solution vector) of the offspring. Finally, after the *Evaluation* of Q(t), the new population P(t+1) is obtained by applying the *Replacement* operator. In order to avoid population stagnation and preserve its diversity and entropy, we have selected the best individual in Q(t) to replace the worst one in P(t) (Stolfi et al., 2020a, Stolfi et al., 2020d) if it has a better fitness value.

Each individual is evaluated against a number of best opponents (up to 30 in our study) which are taken randomly from its adversary’s Hall of Fame. The Hall of Fame of predators is populated with the best individual of GAU after each generation avoiding repetitions. The Hall of Fame of preys follows the same policy using the best escaper from GAE.

When the maximum number of evaluations is reached, a master tournament is conducted in which predators and preys from their respective Hall of Fames are faced each other in order to obtain the best specimens of each side, becoming in this way the solutions (predator and prey configurations) calculated by each CompCGA’s run. As this is a stochastic algorithm we perform 30 independent run for each case study (described in the next Section). Table 2 summarises the parameters of the proposed CompCGA.

**TABLE 2:** Parameters of the proposed CompCGA. *L* is the length of the configuration vector.

Parameter	Value
Population (μ)	20
Offspring (λ)	20
# Evaluations	20,000
Crossover probability ($P_{c}$)	1.0
Mutation probability ($P_{m}$)	1/L

### 3.4 Case Studies

Four case studies are analyzed in this article comprising 2, 4, 8, and 16 escapers which try to run away from a restricted area. The surveillance system is made of four swarms of 4 UMVs, four swarms of 4 UAVs, and one swarm of 2 UGVs, i.e. 34 autonomous vehicles in total.

**FIGURE 5:**
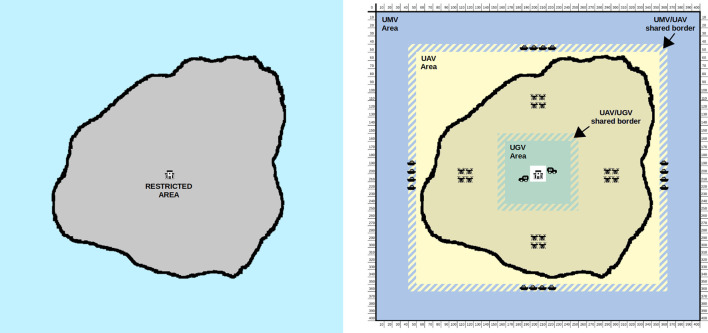
case study. **Left:** the surveillance geographic area. **Right:** the different vehicle swarms and their assigned area in the surveillance scenario. Vehicles’ shared areas are rayed.

The analyzed scenario is set up in a fictional island having a restricted area in its center (Figure 5). Three patrolling areas were defined, the innermost for UGVs where flying devices are not allowed for safety reasons. The central area is assigned to UAVs, which are faster and have cameras pointing toward the ground, and finally, the outer patrolling area comprises water surface so that UMVs are the last detection barrier to be defeated by escapers. There are shared borders between patrolling areas, i.e. 1-m wide regions where both type of vehicles can coexist, to easy the transition between different type of swarms. Since those vehicles are moving at different altitudes (UMV/UAV and UAV/UGV) there is no risk of collision in such shared areas.

The map dimensions are 400 × 400 m, the simulation time was set to 10 min and the escapes can take place during the time slot going from 100 to 400 s. The bottom limit is for allowing the initial spread of the swarms and the top limit is to give enough time to escapers to reach the border of the map.

### 3.5 Evaluation

**FIGURE 6:**
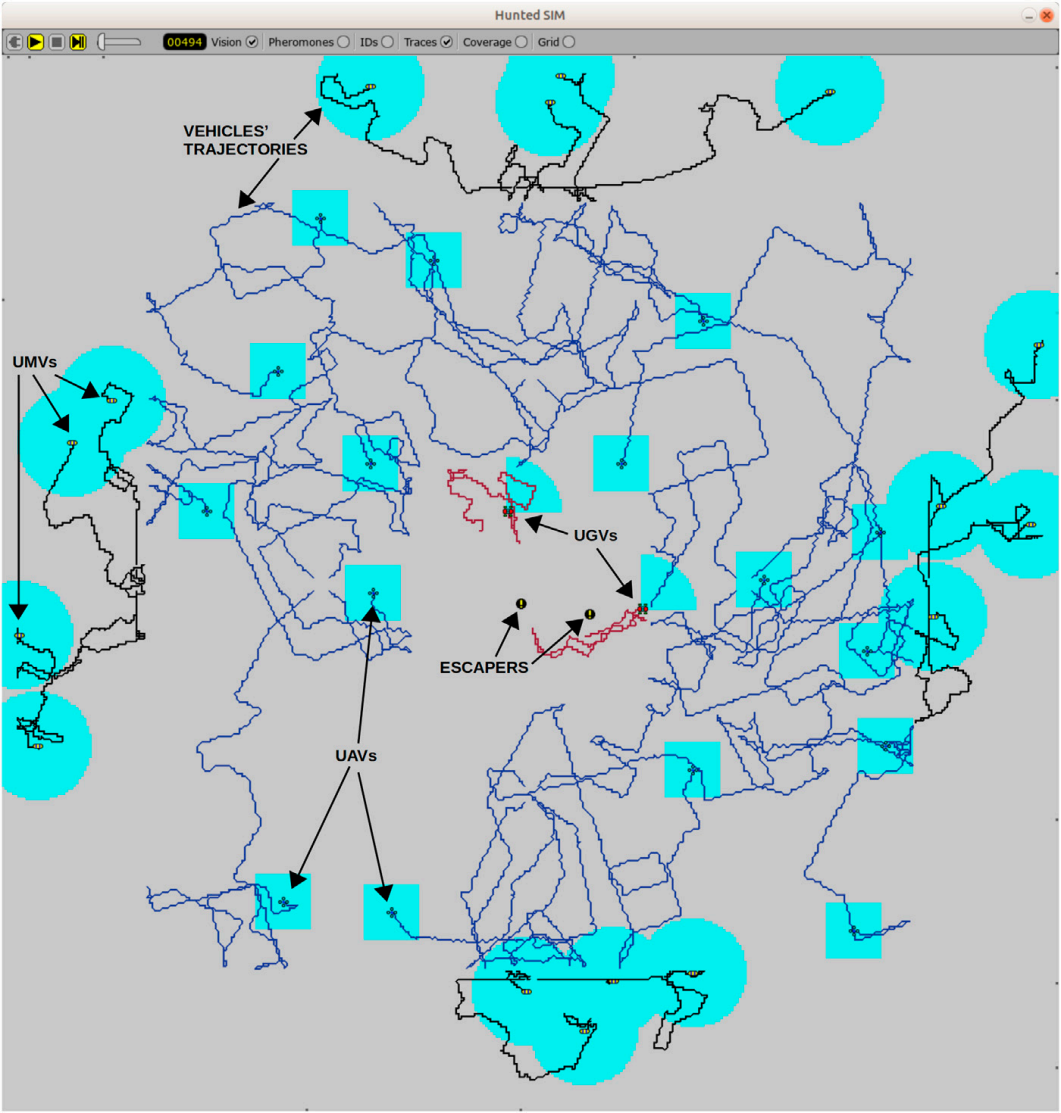
Snapshot of the Hunted Sim where each swarm is spread by the surveillance map. Vehicles trajectories can be seen as well as their detection area. Near to the central area, two escapers are starting their run away.

In order to evaluate each individual representing the configuration of predators and preys we use the Hunted Sim (Stolfi et al., 2020b, Stolfi et al., 2020d). Hunted sim is a simulation environment dedicated to simulate diverse unmanned vehicles in different scenarios involving not only escapers but also intruders. It considers a map divided in 1 × 1-m cells by which vehicles move following a mobility model (Figure 6).

Each individual is represented by a vector $\vec{x}$ (Eq. 1) containing the configuration of the N=34 predators and the *M* preys (M∈{2,4,8,16}, depending on the case study). The evaluation of $\vec{x}$ using the simulator and the fitness function (Eq. 2) provides the fitness value of each individual. The fitness function averages the detection distance (δ) of each escaper spotted and adds a penalisation constant ω for each missing escaper (η). The value of ω is the maximum distance from the map center to its corners. It is calculated according to Eq. 3 where $D_{x}$ and $D_{y}$ are the width and height of the map. Predators (GAU) focus on minimising the detection distance and detecting a maximum number of escapers, consequently, the lower the value of $F\left(\vec{x}\right)$, the better. On the opposite side (GAE), the preys’ objective is to maximise its fitness value by being detected as far as possible from the restricted area or even reaching the border of the map if possible.

$$\vec{x}=\left\{r_{1},\ldots ,r_{N},\varepsilon_{b_{1}},\varepsilon_{c_{1}},\varepsilon_{s_{1}},\varepsilon_{t_{1}},\varepsilon_{a_{1}},\ldots ,\varepsilon_{b_{M}},\varepsilon_{c_{M}},\varepsilon_{s_{M}},\varepsilon_{t_{M}},\varepsilon_{a_{M}}\right\},$$ (1)

$$F\left(\vec{x}\right)=\bar{\delta }+\omega \times \eta ,$$ (2)

$$\omega =\frac{1}{2}\sqrt{D_{x}^{2}+D_{y}^{2}}.$$ (3)

## 4 Results

The experimentation done consisted of the optimisation of CROMM-MS using the CompCGA followed by the analysis of the results obtained including surveillance metrics for each case study, and the comparison with CROMM. Note that CROMM was not optimised since it does not present any parameter.

Thirty runs of CompGGA were performed where the parameters of each vehicle in the swarm were optimised to maximise the escaper’s detection rate using the evaluation function discussed in Section 3.5. Table 3 shows the fitness of the best individual (predator specimen) after performing the master tournament between the configurations stored in both Hall of Fames (predators vs. preys). Additionally, the fitness values for CROMM were obtained to test this mobility model and know how it performs against the best escapers (preys). All in all, CROMM-MS has achieved lower (better) fitness values on average and best values than CROMM in the four case studies analyzed. CROMM-MS has achieved an average improvement of 38.4% over CROMM, and the best fitness value is 41.2% better when using our proposal in the four case studies. These results has a statistical significance greater that 95% (greater that 99% for 4, 8, and 16 escapers) which has been calculated using the Wilcoxon test.

**TABLE 3:** Optimisation results for CROMM-MS after performing master tournament between the best prey and predators obtained from the 30 independent runs of CompCGA. Additionally, results of CROMM are also provided.

	Fitness values						Wilcoxon
	CROMM		CROMM-MS		Improvement		
# Escapers	Average	Best	Average	Best	Average	Best	*p*-value
2	289.343	118.292	**233.603**	**58.292**	19.3%	50.7%	0.047
4	354.785	134.773	**267.587**	**81.715**	24.6%	39.4%	0.006
8	598.123	171.215	**354.673**	**98.463**	40.7%	42.5%	0.000
16	703.281	145.598	**341.886**	**96.854**	51.4%	33.5%	0.001
Average	486.383	142.470	**299.437**	**83.831**	38.4%	41.2%	—

Best results are in bold.

**FIGURE 7:**
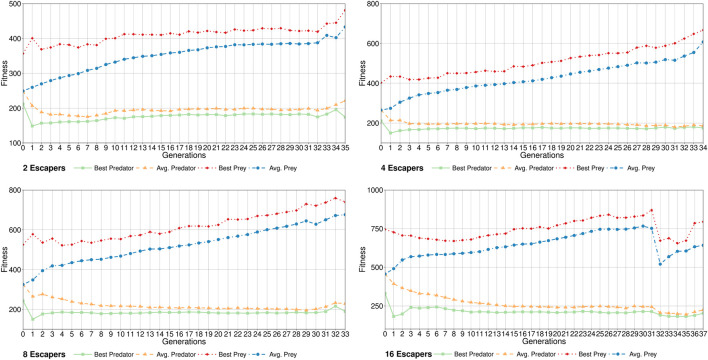
Average convergence of Predators and Preys of the 30 runs of CompCGA for each case study.

Figure 7 shows the average evolution of fitness values for the 120 runs (30 per case study) of CompCGA. It can be seen that initially both populations have approximately the same average fitness value which is evolving to lower values (minimisation) for predators and higher values (maximisation) for preys. In the case study with 16 escapers a sharp change in this tendency is observed in generation 31 where the GAU (predators) have found a new good configuration for vehicles hard to beat by the preys. Note that all the best individuals collected from each generation are stored in their respective Hall of Fame to be used in the final master tournament.

As the number of escapers in a real world scenario is *a priori* unknown, we have taken the best predators from the optimisation process (of each case study) and tested them in all our case studies as shown in Table 4. By doing so, we have chosen the configuration for the vehicles which is the most successful not in one but in the four different case studies proposed in our approach. The results show that although each configuration achieved the best results for its own case study, the configuration for 16 escapers turned out to be the best on average.

**TABLE 4:** Results for CROMM-MS after testing each best configuration obtained in the four case studies (2, 4, 8, and 16 escapers).

	CROMM-MS			
# Escapers	2	4	8	16
2	**233.6**	291.2	324.5	286.9
4	294.5	**267.6**	285.4	318.8
8	430.7	367.7	**354.7**	405.6
16	605.8	588.7	637.0	**341.9**
Average	391.1	378.8	400.4	**338.3**

Best individual results and the best average value are in bold.

Based on these results we analyze the other metrics of the system using the configuration for 16 escapers. Table 5 shows these metrics where the surveillance results of CROMM-MS are compared with CROMM. It can be seen that not only the former achieved a better detection rate (88.8% vs. 84.3% on average), but also detections occurred closer to the restricted area (171 vs. 180 m on average). Furthermore, the area covered by the autonomous vehicles using CROMM-MS was greater (89.2%) than when using CROMM (82%). All these results plus the statistical tests provided (in terms of fitness values), confirm that our approach using a fine parameterisation effectively improves the performance of a heterogeneous multi-swarm surveillance system.

**TABLE 5:** Metrics obtained from the surveillance results of CROMM-MS compared with CROMM. CROMM-MS was configured with the best configuration achieved for the case study with 16 escapers.

	Coverage (%)		Detections (%)		Avg. Distance (m)	
# Escapers	CROMM	CROMM-MS	CROMM	CROMM-MS	CROMM	CROMM-MS
2	82.0	**89.2**	**83.3**	**83.3**	195.1	**192.6**
4	82.0	**89.2**	84.2	**86.7**	175.7	**168.0**
8	82.0	**89.2**	81.3	**89.2**	173.9	**160.5**
16	82.0	**89.2**	88.3	**96.0**	175.3	**162.8**
Average	82.0	**89.2**	84.3	**88.8**	180.0	**171.0**

Best results are in bold.

**FIGURE 8:**
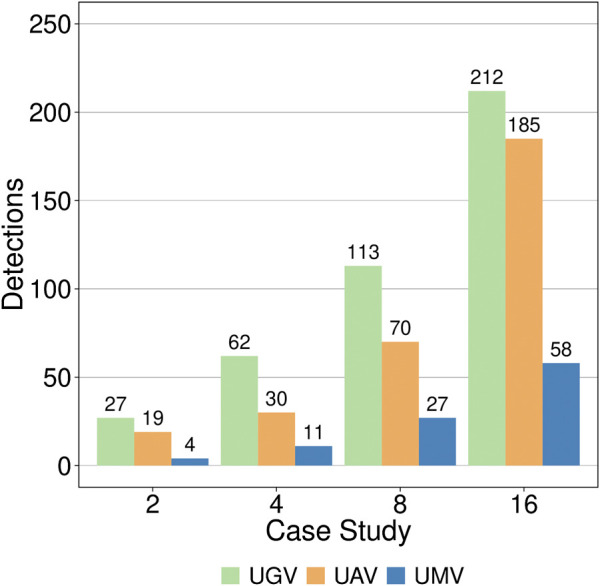
Escaper detections grouped by case study and vehicle type.

Finally, a last study regarding the number of detections arranged by vehicle type was done. It can be seen in Figure 8 that UGVs are spotting the majority of escapers as they are the first obstacle to be overcome and our evaluation function prioritises early detections. In the midfield UAVs are doing almost the rest of detections and the last barrier, i.e. UMVs, make the rest of detections. This last study supports the idea of using multi-swarms of vehicles and several patrolling areas as a viable surveillance system in which each vehicle’s characteristics are exploited to improve the system efficiency as a whole.

## 5 Discussion

In this paper we presented a surveillance system based on a new mobility model called CROMM-MS (Chaotic Rössler Mobility Model for Multi-Swarms) with the aim of patrolling and detecting individuals escaping from a restricted area.

We have proposed the parameterisation of CROMM (Chaotic Rössler Mobility Model) in order to address heterogeneous multi-swarms (UAVs, UGVs and UMVs) where early detection has priority over coverage. A new Competitive Coevolutionary Genetic Algorithm (CompCGA) was designed to optimise vehicles trajectories as well as escapers’ evasion ability using a predator-prey approach.

The results obtained after 30 independent runs of CompCGA for four case studies (34 autonomous vehicles vs. 2, 4, 8, and 16 escapers) show that CROMM-MS has successfully detected 89% of escapers, performing better than CROMM (84%), not only in terms on early detection of escapers, but also in area coverage (89% vs. 82%).

As a matter of future work, we would like to improve our system even more using other techniques for spreading the swarm such as virtual pheromones or ghost vehicles, increasing the detection rates as well as area coverage. Moreover, we believe that CROMM-MS could be also adapted to detect intruders trespassing a restricted area. Consequently, we would like to try our approach in this kind of scenarios as well as different map sizes, geographical characteristics as well as using different swarm members. Despite the competitive results achieved by CompCGA, we would like to test different optimisation approaches for our surveillance system, e.g. Differential Evolution (DE) and Particle Swarm Optimisation (PSO).

## Data Availability

The raw data supporting the conclusions of this article will be made available by the authors, without undue reservation.
